# Más allá de lo fabril: la potencia de los territorios

**DOI:** 10.18294/sc.2025.5968

**Published:** 2025-12-01

**Authors:** Hugo Spinelli

**Affiliations:** 1 Doctor en Salud Colectiva. Investigador, Instituto de Salud Colectiva, Universidad Nacional de Lanús, Buenos Aires, Argentina. hugospinelli09@gmail.com Universidad Nacional de Lanús Instituto de Salud Colectiva Universidad Nacional de Lanús Buenos Aires Argentina hugospinelli09@gmail.com

**Keywords:** Análisis Institucional, Deshumanización, Industrialización, Territorio, Institutional Analysis, Dehumanization, Industrialization, Territory

## Abstract

El artículo propone una crítica a la hegemonía del modelo fabril como matriz organizativa de las instituciones sociales. Ese modelo fabril ha obturado poder entender la complejidad ontológica de lo social y la singularidad de los territorios. A partir de una articulación teórica que incluye aportes de Nietzsche, Spinoza, Bourdieu, Elias, Santos, Deleuze y Guattari, así como tradiciones de la geografía crítica y el análisis institucional, el texto argumenta que la adopción acrítica del paradigma industrial deshumaniza y fragmenta los problemas sociales, favoreciendo la reproducción de las desigualdades sociales. Se señala cómo, en el siglo XIX, las vinculaciones entre Estados y profesionales se dieron en el marco de “procesos de integración y de diferenciación”, que no incluyeron a los territorios. Frente a ello, se reivindica la necesidad de diseñar institucionalidades pequeñas, rizomáticas, relacionales y lúdicas, capaces de dialogar con saberes situados y fortalecer procesos de publicización de los problemas sociales en los territorios. El artículo concluye que transformar los campos sociales requiere abandonar el imaginario fabril, recuperar el carácter artesanal del trabajo social y construir instituciones ancladas en la potencia de los territorios, proponiendo que “del laberinto se sale por arriba”.

## Abstract (en)

This article offers a critique of the hegemony of the factory model as the organizational matrix of social institutions. This factory model has hindered an understanding of both the ontological complexity of the social world and the singularity of territories. Drawing on a theoretical articulation that includes contributions from Nietzsche, Spinoza, Bourdieu, Elias, Santos, Deleuze and Guattari, as well as traditions from critical geography and institutional analysis, the text argues that the uncritical adoption of the industrial paradigm dehumanizes and fragments social problems, reinforcing the reproduction of social inequalities. It examines how, in the nineteenth century, the relationships between states and professionals developed within “processes of integration and differentiation” that excluded territories. In contrast, the article underscores the need to design small-scale, rhizomatic, relational, and playful institutional arrangements capable of engaging with situated forms of knowledge and strengthening processes of publicizing social problems within territories. The article concludes that transforming social fields requires abandoning the factory imaginary, recovering the artisanal character of social work, and building institutions rooted in the power of territories, proposing that “the way out of the labyrinth is from above.

## Introducción

“si los individuos definen las situaciones como reales, son reales en sus consecuencias” Teorema de Thomas^1^.

Durante la modernidad, mientras los modelos fabriles se multiplicaban, el mundo académico hegemónico ignoraba las singularidades y complejidades de los territorios. Y en vez de subordinar las instituciones sociales a los territorios, subordinó los territorios a los modelos fabriles que fueron adoptados por las instituciones sociales^2^^,^^3^^,^^4^. Esa situación, aún vigente, se sustenta en cuatro premisas que articulan este trabajo:

- El imaginario social sobre lo institucional está dominado por las grandes instituciones industriales, de allí que los edificios de estas instituciones tiendan a imitar, en sus tamaños, a las grandes fábricas. El uso del adjetivo “grande” hace referencia a lo tangible, es decir, a las características edilicias y materiales de la institución y no a su dimensión simbólica.
- La geografía física trabajó y se limitó a la idea de lugar, sin incluir lo social. Sin embargo, la geografía social diferenció los conceptos de lugar y territorio, considerando a este último como un espacio social habitado, producto de una construcción social que integra sistemas de objetos y de acciones, donde el tiempo y la interacción humana son fundamentales^5^^,^^6^^,^^7^.
- Las instituciones expresan significaciones, normas, valores y prácticas que se enmarcan entre lo normativo y lo simbólico, orientando la vida institucional, que es la forma concreta y sedimentada que adquieren los actores, en un equilibrio dinámico entre conservación y cambio, el cual es presentado y entendido, mayoritariamente, como “natural”^8^^,^^9^.
- Las complejidades de lo institucional en lo social y sus relaciones con los territorios, en general, no son problematizadas, por ello vamos a sostener la necesidad de su publicización, es decir, hacer público los problemas señalados para que formen parte de la agenda social y política^10^^,^^11^.

En síntesis, este trabajo critica la naturalización de la impronta fabril en las instituciones sociales, que niega su ontología social^12^. 

Para discutir estas ideas nos preguntarnos: ¿el modelo fabril es el modelo institucional a imitar en los campos sociales?, ¿las diseños institucionales se adecuan al carácter ontológico de lo social y a la complejidad de los problemas que se enfrentan?, ¿cuáles son los beneficios de esas grandes instituciones para los territorios?, ¿las disciplinas universitarias hegemónicas entienden la complejidad de los territorios?, ¿qué debiera caracterizar a las instituciones de los campos sociales desde una concepción territorial?^13^^,^^14^. Nuestra hipótesis es que los diseños fabriles deshumanizan y anulan lo relacional y lo múltiple, cosificando a los territorios, a sus poblaciones y a los trabajadores y las trabajadoras de diferentes campos sociales que trabajan en ellos^15^. Por lo tanto, proponemos centrar las acciones en los territorios, desde múltiples y pequeñas instituciones, que respondan a las preguntas, y a los problemas de esas poblaciones con el firme propósito de reducir desigualdades sociales. 

## Lo fabril en lo social

Para la tradición filosófica clásica, la verdad es el descubrimiento de lo real, en tanto producto de la relación entre el pensamiento y la realidad. La verdad así concebida, es presentada como absoluta y universal. Nietzsche, en cambio, la postula como invención de sentidos que representan perspectivas y fuerzas vitales que él relaciona con convenciones sociales, en tanto acuerdos prácticos que facilitan la convivencia, mientras se mantengan como verdades^16^^,^^17^^,^^18^. Nietzsche considera la verdad como “un ejército móvil de metáforas”. Para él no existe una única verdad absoluta, sino que hay interpretaciones que surgen desde perspectivas vitales e históricas, que no reflejan el mundo, sino que le imponen un sentido al mundo, que es el producto de luchas históricas. En el juego social se producen múltiples interpretaciones sobre los hechos, algunas más potentes que otros, lo que expresa que el mundo no es estable, y que el conocimiento es una expresión de la voluntad de poder, donde no se busca la verdad, sino que se busca ejercer poder, ordenar, dar forma, y dominar el caos^16^^,^^17^^,^^18^. 

Con Nietzsche, la verdad deja de ser absoluta y metafísica, para pasar a ser una construcción humana, ligada a la vida, a la ilusión, y a la metáfora. En sus textos. Nietzsche considera la verdad como una convención útil, pero que no refleja la realidad “tal cual es”. Para él, lo importante no es la verdad en sí, sino qué efecto tiene sobre la existencia de las personas, si afirma, potencia y da forma a la vida, o si la niega y debilita^16^^,^^17^^,^^18^. Las ideas de Nietzsche sobre la verdad, se ubican por fuera de la tradición filosófica racionalista/idealista, y tienen influencia en el pensamiento de Heidegger^18^, Gadamer^19^, Foucault^20^, Bourdieu^15^, y Badiou^21^, entre otros. 

Concebir la verdad como una construcción social permite problematizar lo que es presentado como “natural” por la ciencia dominante, y que en este texto esa verdad es representada por el modelo fabril, en tanto modelo institucional para los campos sociales. Es esa verdad impuesta la que nos proponemos desnaturalizar. 

## La orfandad de teorías organizativas

El feudalismo agrario fue la forma productiva dominante durante la Edad Media, desde el siglo V hasta el siglo XV. Recién entre los siglos XIII y XV se produce el crecimiento de las ciudades, del comercio y de la artesanía urbana, y se desarrollan otras producciones mercantiles y artesanales. Con el inicio del capitalismo en el siglo XV, y durante la Revolución Industrial a mediados del siglo XVIII, se producen grandes transformaciones económicas, urbanas, laborales, y ambientales, que repercuten en los modos de vida. Durante esas transformaciones, la fábrica pasó a ser el lugar del trabajo y el lugar para las máquinas, a la vez que se abandonaron los talleres artesanales. 

El término fábrica pasó con el tiempo a asociarse a la fabricación de productos. Y, si bien, en la actualidad los términos artesano y fábrica se entienden de maneras muy diferentes, no siempre fue así, como surge al analizar las etimologías de ambos conceptos:

- *Artesano*: proviene de arte. “Conjunto de preceptos para hacer bien algo”, del latín ARS, ARTIS, f., “habilidad”, “profesión, arte”^22^.
- *Fábrica*: tomado del lat. *fabrica “*oficio de artesano”, “arquitectura”, “acción de labrar o componer”, “taller”, “fragua”, abreviación de *ars fabrica “*arte del obrero o artesano”, derivado de *faber,* que en latín designa a este último^22^.

Richard Sennett, defensor de lo artesanal^23^, recupera un relato crítico sobre la industrialización durante el siglo XX en Moscú, allí Walter Benjamin describe:

…una fábrica modelo que producía bramante y bandas elásticas, cuyos trabajadores eran en su mayor parte mujeres de edad mediana. A la vera de las operarias yacían las máquinas modernas, apagadas por falta de piezas, mientras aquellas trenzaban las hebras a mano igual que un siglo antes. Sin embargo, la fábrica era cien veces más grande que los talleres en los que esta artesanía se había practicado en otra época, una gran caja moderna en la que lo “moderno” era una categoría vacía.^24^

Durante el siglo XX, la hegemonía institucional de la fábrica se tornó absoluta, y su importancia se consideró proporcional a su tamaño, omitiendo que al interior de esas instituciones trabajaban personas en el marco de relaciones sociales que fueron negadas y cosificadas. 

Lo industrial se originó en el capitalismo, sin tener una teoría organizativa propia, la cual recién se consiguió entre fines del siglo XIX e inicios del siglo XX. Es decir que al capitalismo le llevó dos siglos tener una teoría organizativa acorde con el modelo productivo. Mientras tanto, suplió esa carencia con la adopción de las formas organizativas de la iglesia y el ejército, que eran instituciones milenarias con estructuras organizativas que precedieron al capitalismo^25^. Esa ausencia de teoría se subsana recién a fines del siglo XIX y principios del siglo XX, cuando Frederick Taylor (1856-1915), ingeniero mecánico, y Henri Fayol (1841-1925), ingeniero de minas, proponen formas organizativas para el trabajo y la producción industrial^26^^,^^27^. Ambos parten de la premisa de concebir al trabajador como una máquina, siguiendo las ideas de René Descartes sobre el cuerpo humano^12^. 

Taylor estudió el proceso de trabajo y los movimientos del trabajador desde una perspectiva mecánica, en tanto Fayol trabajó sobre las estructuras y las funciones de la organización para alcanzar la eficiencia. Ambos, de manera independiente, plasmaron las bases interpretativas de lo pasó a conocerse como *teoría general de la administración* (TGA) la cual representa el pensamiento administrativo/industrial desde inicios del siglo XIX hasta la actualidad^26^^,^^27^^,^^28^. 

En su devenir, la fábrica se constituyó en una institución paradigmática para el disciplinamiento de los individuos^25^, tanto en el capitalismo como en el socialismo. De hecho, el socialismo consideró que al haber resuelto el tema de la propiedad de los medios de producción podía incorporar acríticamente el taylorismo y el fayolismo. Las consecuencias de esas decisiones las padecieron los trabajadores, que se alienaron, producto de la simplificación interpretativa realizada por el socialismo sobre la fuerza de trabajo. 

Desde sus inicios, la TGA fue, y sigue siendo, funcional a la lógica del capital y al desarrollo industrial, sobre la base del pragmatismo. Con el tiempo, se integró a diferentes teorías y disciplinas, sin perder su relación con la acumulación del capital.

Luego de la Segunda Guerra Mundial, en el año 1942, William Beveridge (1879-1963), economista liberal inglés, propuso políticas sociales caracterizadas por una fuerte intervención del Estado, ideas que plasmó en su libro *Social Insurance and Allied Service*^29^. Allí planteó compensar las consecuencias sociales del avance del capitalismo, para así detener la amenaza del comunismo en la posguerra. Beveridge planteó diferentes acciones para reconstruir Europa, entre las que destacó, la de destruir “la indigencia, las enfermedades, la ignorancia, la suciedad y la ociosidad”, a las que llamó los cinco males gigantes^29^. Varias décadas después, en 1995, Robert Castel publicó *La metamorfosis de la cuestión social*^30^, donde sostuvo que la cuestión social era “una aporía fundamental sobre la cual una sociedad experimenta el enigma de su cohesión y trata de conjurar el riesgo de su fractura”^30^. Al usar el concepto de aporía, Castel señala en la “cuestión social” contradicciones insolubles, paradojas y enunciados inviables de carácter racional, que lo llevan a adjetivarla de aporía. Esto nos permite entender no solo el devenir del Estado benefactor, sino también la persistencia de “los cinco males gigantes”, que Beveridge se había planteado destruir^29^. 

En ese contexto, el “esplendor” de lo industrial anuló la posibilidad de poder pensar otra institucionalidad para los campos sociales, a los que se les impuso como “verdad”, la teoría organizativa de la lógica industrial, tanto en la concepción del trabajo, como en sus formas de dirección. Así las instituciones sociales pasaron a ser entendidas como espacios en los que se replicaba el modelo fabril, situación que persiste hasta nuestros días. 

Las instituciones sociales (centros de salud y hospitales; escuelas, institutos y universidades; juzgados y cárceles; diferentes servicios sociales, etc.), junto con los problemas sociales (la explosión urbana, la falta de viviendas, las inequidades, el deterioro del ambiente, la pobreza, la indigencia, la delincuencia juvenil, las cuestiones raciales y de género, el trabajo infantil, las adicciones, y la discriminación), ponen en evidencia que el modelo de institución para trabajar esos problemas no es el fabril. Esto no constituyó ni constituye un problema para las lógicas dominantes, que pensaron y piensan en políticas sociales asistencialistas y/o focalizadas y no en políticas originadas desde los territorios, que acompañen la construcción de ciudadanía social en los grupos vulnerables, ni en reducir las desigualdades sociales^31^^,^^32^, y mucho menos hacer que esos problemas sociales se tornen problemas públicos, es decir, publicizarlos^10^^,^^11^ para construir consenso sobre la necesidad de ser abordados.. 

## Dividir los problemas sociales

Durante la modernidad, los diseños y el funcionamiento de las instituciones sociales llevaron a que la mayoría de los problemas sociales se transformara en nudos gordianos, que aún hoy deben ser enfrentados por quienes trabajan y gestionan en las áreas sociales^33^^,^^34^. 

La razón moderna no solo les negó a las instituciones sociales su ontología, sino que las subordinó a las lógicas racionales del mundo industrial, dominadas por la relación *Homo sapiens/Homo faber*, desterrando el juego al olvido, por no ser funcional a la lógica del capital, cuando hasta los inicios de la revolución industrial lo lúdico había estado presente en diferentes épocas y lugares^12^^,^^35^. 

En 1904, Max Weber caracterizó la burocratización de las sociedades capitalistas occidentales como un producto de la racionalización de la vida social, a la que entendía como un sistema basado en la eficiencia teleológica, el control y el cálculo racional. Talcott Parsons, en tanto traductor al inglés de los textos de Weber, creó en su traducción la metáfora de la “jaula de hierro” para describir la racionalización que, según Weber, atrapaba tanto a quienes administraban como a quienes dependían de ella^33^. 

Desde hace décadas, ese proceso de racionalización acompaña la crisis del hombre público, producto del desequilibrio entre la vida pública y la vida privada^36^^,^^37^^,^^38^. Este proceso se incrementó en los últimos años con el surgimiento en diferentes países de gobiernos que tienen como propósito acabar con las instituciones públicas, o reducirlas a su mínima expresión, y para ello fomentan su descrédito, al que adhieren los sectores populares ante la falta de respuestas a sus demandas y necesidades desde las instituciones sociales del Estado^39^^,^^40^. Ese descrédito de lo público es producto de múltiples situaciones, como la crisis de representatividad del Estado; los avances del neoliberalismo; la anulación epistemológica de la otredad y lo relacional, la hegemonía de una cultura dominante que renunció a conformar sociedades menos desiguales^36^^,^^37^^,^^38^; de Estados sin ciudadanos^31^, y de la profundización de la mimetización de lo social con la lógica industrial. Todo ello produjo y produce en la sociedad y al interior de las instituciones más desencuentros que encuentros, y más órdenes que conversaciones. Todo ello dificulta construir lazos sociales fuertes, entre los trabajadores y los territorios^15^. 

Las instituciones sociales, al divorciarse de las necesidades y demandas de los territorios, asumieron un cientificismo^41^ -ciencia descontextualizada- basado en tres premisas de la modernidad: el sujeto autoconsciente, que sostiene la noción del progreso en el conocimiento; la posibilidad de conocer el objeto exterior al sujeto; y el poder dar cuenta de las leyes de funcionamiento del objeto para apropiarse de la realidad^3^^,^^42^. Esas premisas crearon y continúan creando la ilusión de poder controlar y planificar el mundo, en función de los conceptos sobre los que descansa el proyecto de la modernidad: la razón, el progreso, la unidad, la totalidad, la representación, la conciencia, la posibilidad, la ciencia, la felicidad y el hombre, entre otros. Esos conceptos fueron y siguen siendo el sustento de diferentes desarrollos teóricos que sostienen desde hace cinco siglos el proyecto de la modernidad que pretende alcanzar un orden que no es propio ni de lo social, ni de lo territorial, sino de lo industrial^2^^,^^3^^,^^12^^,^^42^. Sobre las ideas de lo industrial se constituyó el imaginario de que las instituciones, cuanto más grandes, son mejores, lo que llevó y lleva a la deshumanización, que alcanza su máxima expresión en las “instituciones totales”, en las que es muy difícil poder diferenciar al trabajador del paciente, en el caso de los hospicios, o al detenido del que vigila en la cárcel^43^.

La irónica frase de Robert Castel^30^ sobre “dividir la cuestión social en tantas partes como sea necesario para eludirla mejor”, parodia la lógica del método cartesiano, y sintetiza lo inútil que resulta buscar, en el racionalismo y sus vertientes, los marcos referenciales del hacer-pensar de las instituciones sociales del Estado. Es indudable que se necesitan otras epistemologías^44^^)^ , que incluyan además una vigilancia epistemológica de los propios sujetos sobre sus pensamientos y acciones^15^, ya que la naturalización de esas omisiones epistemológicas impiden un hacer/pensar diferente que nos traicionan sin avisarnos. 

## Estados, profesionales y la negación de los territorios

Para Norbert Elias (1987-1990), en el siglo XIX, las sociedades organizadas como Estados se conformaron por grupos básicamente aristocráticos preindustriales, que hacían de esos Estados un objeto de identificación colectiva, lo que habilitaba a su interior el desarrollo de la economía como una nueva ciencia, desde la cual se exigía libertad económica para las empresas, y se limitaba la intervención del Estado^45^. Así, las vinculaciones entre Estados y profesionales se dieron en el marco de “procesos de integración y de diferenciación”, que no incluyeron a las poblaciones a las cuales nos referimos con “la negación de los territorios”^45^.

Desde un punto de vista sociológico, el desarrollo de la organización político-estatal y las posiciones de los profesionales son aspectos indivisibles de la transformación del marco social contemporáneo, que no hacen más que señalar los aspectos de diferenciación e integración que se produjeron durante la conformación de los Estados modernos, momento en que el acceso y la ocupación de diferentes posiciones sociales confirió a sus portadores potenciales oportunidades de poder^45^. Elias considera que uno de los problemas centrales de las sociedades altamente diferenciadas es el control institucional eficaz de todas las posiciones sociales de coordinación e integración, y se pregunta “¿qué posibilidades hay de asegurar socialmente que los que detentan posiciones de poder, releguen parte de sus intereses en función de un bien mayor para la sociedad?^45^. La pregunta que se formula Norbert Elias mantiene total vigencia.

En los procesos de industrialización, las vinculaciones entre Estado y profesionales se dieron en el marco de relaciones que disputaron precios y tasas de ganancia sobre las mercancías y los servicios. Esas lógicas, al ser llevadas mecánicamente a los campos sociales, provocaron la cosificación de las relaciones sociales, negando el carácter ontológico de lo social^45^, 

Para Elias, la dinámica social remite a procesos que implican alcanzar, reducir o perder posiciones sociales^45^. Así, los actores que participan de la integración entre Estados y profesionales, buscan ocupar posiciones, mientras los agentes que permanecen por fuera de esas integraciones tratan de superar los olvidos y negaciones que han recibido históricamente, mientras persisten, o abandonan, sus luchas para que se reconozcan sus derechos^45^. Es muy necesario que, a esa vinculación entre Estado y profesionales, se sumen los intereses de los conjuntos sociales de los territorios, para discutir con ellos sus problemas, y recuperar otras miradas y experiencias, con el propósito de construir consensos que vayan más allá de lo económico y las tasas de ganancia^45^. Ello será imposible si no se reconoce, se discute y se asume el giro corporativo que tomaron las instituciones sociales, dándole las espaldas a los territorios y a las instituciones.

## Los juegos de los agentes sociales en los territorios

Los beneficios del Estado benefactor, propuesto por Beveridge, se fueron deteriorando en el tiempo por diferentes circunstancias, lo cual abonó las críticas que señalaban la progresiva separación entre el Estado y las necesidades sociales. Esa crisis del rol del Estado frente a los problemas sociales evidenció diferentes dinámicas del juego social, que vamos a trabajar desde distintos agentes: las burocracias de calles^46^, la mano izquierda del Estado^47^^,^^48^^,^^49^^,^^50^, las formas de democracias directas^51^^,^^52^^,^^53^^,^^54^, la acción de las iglesias^55^, y la acción social del narcotráfico^56^.

Para Michael Lipsky, el juego de los funcionarios públicos de la base del Estado representa la realidad de los servicios prestados en los territorios. Esa tarea es ejercida por los trabajadores con cierta discrecionalidad, a través de la entrega de bienes o beneficios, y del cuidado del orden público^46^. Lipsky llamó a esos funcionarios del Estado como “burocracias de calles” al estar concentrados en temas de salud, educación y seguridad, esa adjetivación representa un caleidoscopio de situaciones que incluyen: la asistencia social, el control social, la medicalización de la vida cotidiana, disputas de poder, y la corrupción en las acciones sociales, entre otras situaciones posibles. Lipsky concluye que a fin de cuentas, para el Estado -en general- no importa lo que hagan esas burocracias de nivel de calle, ya que le es más fácil y económico crear empleo a ese nivel, que solucionar o reducir los problemas sociales que originan esos empleos^46^^,^^57^.

Pierre Bourdieu formuló los conceptos de “mano derecha y mano izquierda“ del Estado^49^. A la “mano derecha” la describió compuesta por altos funcionarios -tecnócratas que se encargan del diseño y la gestión de las políticas desde perspectivas técnicas-; por el poder económico, el poder judicial y el poder policial; los ministerios de Hacienda y Economía; los bancos públicos y privados; y los gabinetes ministeriales. Y a la “mano izquierda” la describió compuesta por el trabajo de los agentes estatales que implementan las políticas sociales formuladas por la mano derecha^47^^,^^48^. 

Esas dos manos, con integrantes e intereses muy diferentes, suelen enfrentarse al interior de un mismo gobierno, que en distintos países se encuentran gobernados por diferentes ideologías. Un ejemplo es el relato de Carlos Matus, desde su experiencia en Chile como integrante del gabinete de ex presidente Salvador Allende, entre 1970-1973, y las peleas y enfrentamientos al interior del gabinete^34^. El conflicto entre los integrantes de ambas manos limita, no pocas veces, solucionar los problemas sociales a través de las políticas públicas del Estado, lo cual fortalece el descrédito en las comunidades, sobre los gobierno y sus instituciones^50^. 

Ante esas realidades, los sectores más vulnerables de la sociedad reclaman, cada vez con menos paciencia, por la calidad de los servicios que reciben, ¡cuando los reciben! Y comienzan a creer en falsas promesas de gobiernos neoliberales con tintes totalitarios, que no solo no dan respuestas a las viejas demandas, sino que, de manera intencional y explícita, las agravan. 

A partir de las décadas de 1980 y 1990, ante el avance del neoliberalismo, la mano izquierda del Estado se debilitó, se deslegitimó y se desfinanció, a la vez que se fortaleció la mano derecha del Estado. En ese devenir, el Estado fue perdiendo su rol de garante de los derechos sociales^38^. 

Bourdieu reconoce que los trabajadores de la mano izquierda tienen una autonomía relativa en relación con las decisiones políticas centrales, la cual radica en que, al permanecer en contacto directo con las poblaciones, enfrentan situaciones muy singulares de vulnerabilidad, que les exigen acciones, pero a la vez les permiten desarrollar una influencia significativa en la vida social, al distribuir recursos, impulsar acciones y, a veces, legitimar prácticas no diseñadas por la mano derecha. En algunos casos, estos agentes desarrollan una conciencia crítica que “juega” en la implementación de las políticas y/o programas a favor de los beneficiarios, cuestionando y hasta modificando en su accionar órdenes y normativas de la mano derecha. En los países del Sur, esa dualidad estructural del Estado -mano derecha y mano izquierda-, no pocas veces lleva a que sea más débil la presencia de la mano izquierda, que en los países centrales. 

Hugo Chávez, cuando fue presidente de Venezuela entre 1999 y 2013, no lograba que las políticas y acciones de gobierno se materializaran en acciones en los territorios, debido a la burocratización de los procedimientos que entraban en laberintos kafkianos^58^. Ante ello, Chávez implementó “*las misiones*”, que eran dispositivos que articulaban temas sociales específicos con los territorios, sin intermediarios, en la búsqueda de lograr que la decisión política del gobierno se transformara en acciones en los territorios^58^.

Diferentes investigaciones y publicaciones realizadas desde el Sur, realizan propuestas decoloniales a través de las acciones y la potencia de las comunidades de base, los grupos étnicos, los colectivos de mujeres, los colectivos de LGBTBYQ+, los pueblos originarios, y otros movimientos sociales, que muchas veces reemplazan con mayor efectividad lo que hacía o debía hacer la mano izquierda del Estado^51^^,^^52^^,^^53^^,^^54^^,^^59^. Entendemos esas propuestas como aproximaciones a formas de democracias directas. 

Las instituciones sociales, en general, enfocan sus acciones en los individuos, en tanto que, en los territorios, el trabajo con los colectivos no tiene responsables jerarquizados y legitimados, y no pocas veces el abandono de los territorios por parte de los Estados, facilita la acción social del narcotráfico^56^, que con sus singulares modalidades de intervención y cooptación configura sus “políticas sociales”^15^^,^^60^^,^^61^.

En los territorios confluyen diferentes modelos institucionales: los que se originan desde el Estado, los que se impulsan desde los propios territorios, junto a diferentes expresiones religiosas, sociales y políticas que |pocas veces actúan de manera sincronizada. Trabajar en los territorios implica enfrentar una complejidad, no siempre reconocida, que coloca a los trabajadores en dinámicas que combinan lo natural y lo social; lo racional y lo relacional; lo objetivo y lo subjetivo y lo individual y lo colectivo; entre otras tantas posibles combinaciones, que incluso pueden ser más que binarias. Esas situaciones son interpretadas desde la mano derecha del Estado como desordenadas e ineficientes, expresando su incapacidad para interpretar la complejidad de lo social, complejidades que enfrenta la mano izquierda del Estado en los territorios^34^^,^^47^^,^^48^. 

## Burocracias profesionales

Henry Mintzberg (1939-), en 1979, en sus investigaciones sobre las formas organizativas de las instituciones de la sociedad, destaca la complejidad y singularidad de lo que llama burocracias profesionales, diseño organizacional que caracteriza a las instituciones sociales^62^. Las burocracias profesionales se basan en un trabajo poseedor de muchas libertades, que se desarrollan básicamente en el núcleo operativo (la base de la organización), lo que las diferencia radicalmente de las burocracias mecánicas (modelos fabriles). Esas libertades que caracterizan a las burocracias profesionales le otorgan mucha autonomía frente a los gobiernos y sus directrices políticas. En ese sentido recuperamos este diálogo de Carlos Matus con un alto funcionario del gobierno de Salvador Allende, que mantienen total vigencia:

En una reunión en el Palacio de la Moneda en Chile un alto funcionario de gobierno me decía: “el gobierno siente que no hay contacto con la universidad ni hay relación práctica de trabajo con la universidad. Pero la universidad nos pide recursos”. Este problema se debe repetir en todas partes.^63^

A pesar de haber sido descriptas hace casi 50 años, el concepto de burocracias profesionales es poco conocido por quienes dirigen y trabajan en instituciones sociales y/o formulan políticas sociales. Mintzberg, al analizarlas, narra regularidades que son desconocidas por los directivos y trabajadores de esas instituciones, quienes buscan, sin encontrar, lo que caracteriza a la fábrica: la orden que se cumple, las normas, el organigrama, y la planificación, por citar solo algunos componentes de la cultura fabril y que los trabajadores, al no encontrar que se cumplan en su espacio laboral, lo adjudican a la singularidad de “su” institución, o incluso son ellos mismos los que no las cumplen, y se lo adjudican a una picardía personal que les permite ese incumplimiento. Todo ello habilita la frase que he escuchado en muchos países de América Latina: “la única solución en esta institución es poner una bomba con todos adentros”. El desconocimiento del concepto de burocracia profesional termina por habilitar esa solución trágica, que por suerte no se lleva a cabo. 

Las burocracias profesionales tienen diferencias radicales con las burocracias mecánicas (Tabla 1) que, en general, son muy poco conocidas a pesar de las numerosas publicaciones que hay al respecto. Ese desconocimiento trae serias consecuencias en quienes las dirigen, en quienes trabajan, y en los territorios^12^^,^^62^. ¿Por qué se desconoce el concepto de burocracia profesional si tiene varias décadas de ser formulada?, es la pregunta que deberíamos pensar y discutir.

**Tabla 1:** Comparación de las principales características estructurales de las burocracias mecánicas y las burocracias profesionales.

Características estructurales	Burocracia Mecánica	Burocracia Profesional
Síntesis conceptual	Cúpula iluminada con gran poder	Laboratorio de decisiones tomadas en gran parte por la base de la organización
Identidad	Son organizaciones basadas en lo jerárquico y centralizadas	Son organizaciones burocráticas, pero no centralizadas; son altamente descentralizadas en su núcleo operativo.
Coordinación	Estandarización de procesos y supervisión directa	Estandarización de habilidades y el ajuste mutuo entre profesionales
Poder	Está representado por la autoridad formal, reside en la alta dirección y la tecnoestructura, que diseña y controla los procesos	Poder del experto, profesionales altamente capacitados que tienen un control significativo sobre su trabajo y toman decisiones basadas en su experiencia
Especialización	Énfasis en la especialización de tareas y la división del trabajo	Especialización de los profesionales en áreas específicas, en las que cada uno aporta sus conocimientos, habilidades muy complejas, y técnicas simples.
Autonomía	Muy alta en la cúpula de la organización, que decide en base a sus propios intereses	Muy alta en la base de la organización (los profesionales y su poder técnico)
Entorno	Opera en entornos estables y predecibles, busca la eficiencia y la estandarización	Opera en entornos complejos y dinámicos, en los que la flexibilidad y la adaptación son importantes, pero tienden a la estabilidad como institución
Sus productos	Tangibles y contables	Muy difíciles de medir, pueden monitorearse de manera procesual
Ejemplos institucionales	Grandes fábricas y siderúrgicas, instituciones militares y grandes empresas	Universidades, escuelas primarias y secundarias, hospitales, bufetes de abogados, firmas de contabilidad, institutos de investigación, laboratorios farmacéuticos de investigación, autoridades aeronáuticas y organismos técnicos, órganos judiciales y cortes supremas, institutos culturales y artísticos especializados, estudios de arquitectura e ingeniería.

Fuente: Elaboración propia con base en Mintzberg^62^.

Asumir que las instituciones sociales son burocracias profesionales señala una complejidad que no caracteriza a lo industrial, ya que las burocracias profesionales se caracterizan por presentar una combinación de elementos que resultan inexplicables para la lógica fabril. De allí que, al no comportarse como las burocracias mecánicas, las instituciones sociales son ridiculizadas, sin asumir de parte de quienes las ridiculizan su propio desconocimiento sobre esas singulares formas organizativas de las instituciones sociales. 

La complejidad de las burocracias profesionales se incrementa de manera significativa con el tamaño de la institución, dado que potencia la autonomía en el núcleo operativo, autonomía que no solo es aprovechada por los trabajadores, sino por todos los que participan del “juego”.

## Una discusión fuera de agenda

Nuestro onirismo animalizado que es tan fuerte respecto a los animales de gran tamaño no ha registrado los hechos y gestos de los animales minúsculos. Gaston Bachelard^64^

Las críticas al tamaño de las instituciones hegemónicas de los campos sociales, y al control social que ejercen, se encuentran en diferentes autores como Erving Goffman, con su concepto de “institución total” producto de sus trabajos sobre hospitales, prisiones y asilos^43^^,^^65^; en Michel Foucault, en sus análisis sobre cárceles, escuelas y hospitales^25^^,^^66^^,^^67^; en Richard Sennett, con sus planteos contra el gigantismo institucional^24^; en Norval Morris y David Rothman, con las reformas penitenciarias^68^; en Zygmunt Bauman, en sus críticas al tamaño y el control social de las grandes instituciones del Estado benefactor^69^; y en Iván Illich, con sus críticas a las instituciones escolares y de salud que monopolizan saberes y reproducen desigualdades^70^. 

Las instituciones sociales son hipercomplejas^71^, y los problemas sociales que se abordan pueden combinar diferente conceptos y categorías que se expresan de infinitas formas, pero no hay nada que señale la necesidad del gran tamaño de los establecimientos para trabajar lo social. Por el contrario, la adjetivación de grandes es un inconveniente para la resolución de la mayoría de los problemas sociales, incluso, muchas veces la cuestión del tamaño no solo no colabora, sino que terminan por agravar los problemas. 

En su devenir, las sociedades se han complejizado al igual que los problemas sociales, y ante la necesidad y a veces el propósito de garantizar derechos, el gran tamaño de las instituciones sociales genera efectos contrarios a los declamados. Se ha naturalizado que los problemas sociales se deben abordar en grandes establecimientos, lo cual favorece la deshumanización. Así tenemos grandes cárceles, hospitales, escuelas, colegios, universidades y tribunales, por citar solo algunos ejemplos, que reciben críticas al subordinar la institución social al modelo fabril. Por ello proponemos para los campos sociales instituciones pequeñas sin posibilidades de expandirse, pero sí de multiplicarse, de manera que predomine la humanización sobre lo instrumental, en el marco de tareas centradas en el respeto a las identidades, los derechos y la dignidad de los colectivos sociales en los territorios, para desde allí acompañar sus demandas, reclamos y necesidades. 

También es necesario recordar que lo social necesita del juego ligado a la tarea -*Homo ludens*-, pero su habilitación es inversamente proporcional al tamaño de la institución, por eso se lo encuentra mucho más en instituciones de menor tamaño^12^^,^^35^^,^^72^.

No idealizamos que resolver el problema del tamaño de las instituciones signifique acabar con los problemas sociales. Tampoco pensamos que la falta de respuestas adecuadas desde el Estado a los problemas sociales se soluciona mágicamente con la reducción del tamaño de las instituciones. Pero sí sería un cambio muy importante que el número de instituciones pequeñas fuera significativamente superior al de las grandes instituciones, y así el campo de la salud podría tener más centros de salud, o institutos de temas específicos -como centros oftalmológicos o traumatológicos, por citar solo algunos ejemplos- sin la necesidad que las especialidades biomédicas encuentren su institucionalidad solo al interior de los grandes hospitales. 

Por otro lado, la educación superior en Argentina no debería limitarse a la institución universidad, cuando la Ley 24521 de Educación Superior legisla sobre universidades e institutos y reconoce que el sistema universitario nacional está integrado por universidades e institutos universitarios, entendiendo a las universidades como instituciones que desarrollan su actividad en diversas áreas disciplinarias no afines, orgánicamente estructuradas en facultades, departamentos o unidades académicas equivalentes, y a los institutos universitarios como aquellos que circunscriben su oferta académica a una sola área disciplinaria. A pesar de que la ley acepta ambas institucionalidades es muy raro encontrar en el ámbito público institutos universitarios. 

El riesgo de la atomización de los saberes, que podría darse en los institutos se puede compensar con tramos comunes con otros institutos y universidades, como también con la implementación de rotaciones por diferentes modelos institucionales de un mismo campo de problemas y/o saberes, sin que ello afecte la calidad del aprendizaje. En esa misma línea de pensamiento, podemos proponer tener cárceles más pequeñas, también llamadas de proximidad, y disminuir el número de las grandes cárceles^68^^,^^73^, razonamiento posible de extender a las institucionalidades de otros campos sociales. 

Es necesario problematizar y publicizar^10^^,^^11^^,^^63^, la necesidad de instituciones a escala humana, accesibles, y contextualizadas, que respeten las idiosincrasias y singularidades de los territorios, que es desde donde se deben generar y dirigir las acciones que conforman las políticas, cuyos resultados se pueden monitorear desde niveles centrales, para evitar desvíos u omisiones, y hasta para compensar desigualdades, con pequeños grupos de trabajo altamente tecnologizados en los niveles centrales. 

En todo lo expuesto, sostenemos a los territorios como centrales en la construcción de nuevas hegemonías, basadas en instituciones pequeñas que aborden lo social de manera integral y no fragmentada, al contrario de lo que reproducen las lógicas disciplinarias, que dividen a las universidades en facultades, y a estas en cátedras. De allí que concebimos a las instituciones sociales caracterizadas por un crecimiento rizomático, con fuertes vinculaciones entre Estado y territorios. 

En la implementación de alternativas a las institucionalidades hegemónicas en los campos sociales, es necesario recuperar diferentes propuestas, no jerarquizadas, como los “centros de día”, las “casas de medio camino”, los centros culturales, los clubes de barrio, los cuidados domiciliarios, la rehabilitación basada en la comunidad, los grupos de autoayuda, etc., que conforman espacios destinados a personas pertenecientes a diferentes colectivos, o con ciertos padecimientos, que deben ser integradas socialmente, evitando institucionalizarlas y estigmatizarlas en estructuras asilares, en las que devienen objetos. 

## Lo pequeño es hermoso

Ernst Schumacher (1911-1977), intelectual y economista alemán, publicó en 1973 el libro *Small is beautiful*^74^, en el que criticó la orientación de la sociedad centrada en lógicas económicas, en desmedro de las personas^74^. Schumacher consideró que tener instituciones de menor tamaño, no constituía un rasgo negativo ni una subvaloración, sino que las enaltecía por su mayor potencialidad humanista, sin que ello afecte su calidad^74^.

…los principios de la teoría de las “economías de escala”, según la cual con las industrias y compañías sucede igual que con las naciones, que hay una tendencia irresistible, dictada por la tecnología moderna, a tener tamaños cada vez más grandes […] La economía de la permanencia implica un profundo cambio en la orientación de la ciencia y la tecnología. Estas tienen que abrir sus puertas a la sabiduría y, de hecho, incorporar sabiduría en su estructura misma. “Soluciones” científicas o técnicas que envenenan el medio ambiente o degradan la estructura social y al hombre mismo, no son beneficiosas, no importa cuán brillantemente hayan sido concebidas o cuán grande sea su atractivo superficial. Máquinas cada vez más grandes, imponiendo cada vez mayores concentraciones de poder económico y ejerciendo una violencia cada vez mayor sobre el medio ambiente no representan progreso, son la negación de la sabiduría. La sabiduría requiere una nueva orientación de la ciencia y de la tecnología hacia lo orgánico, lo amable, lo no-violento, lo elegante y lo hermoso. La paz, como a menudo se ha dicho, es indivisible. ¿Cómo podría, entonces, construirse la paz sobre una base hecha de ciencia indiferente y tecnología violenta? Debemos procurar una revolución en la tecnología que nos dé invenciones y maquinarias que inviertan las tendencias destructivas que ahora nos amenazan a todos. ¿Qué es lo que realmente necesitamos de los científicos y tecnólogos? Yo contestaría: necesitamos métodos y equipos que sean: suficientemente baratos de modo que estén virtualmente al alcance de todos; apropiados para utilizarlos a escala pequeña; y compatibles con la necesidad creativa del hombre […] Las cosas que realmente sirven para algo no han de hacerse desde el centro, no pueden ser hechas por grandes organizaciones, sino por la gente misma.^74^

Veinticinco años después de la publicación de Schumacher, Steven Gorelick, ciudadano estadounidense de formación hidro-geólogo, publicó *Small is beautiful, big is subsidised*^75^, texto basado en ejemplos y datos que evidencian lo que se nos oculta: la fuerte dependencia de las grandes empresas respecto a subvenciones, ayudas y exenciones fiscales, laborales y ambientales, sin las cuales esas megacorporaciones globales no serían competitivas ni eficientes. También Gorelick resalta la peligrosa destrucción social, económica y ambiental que causan esas empresas, y se pregunta: ¿sabemos cómo nuestros impuestos se destinan a impulsar la concentración económica en las grandes multinacionales?, ¿somos conscientes de cómo esto afecta al tejido económico local, al empleo y a la utilización de los recursos naturales? 

Las instituciones pequeñas debieran descubrir en los territorios lo que permanece oculto para las instituciones oficiales, y que se descubre cuando se accede a ellos, y se revela que lo individual es expresión de lo colectivo, y que muchas veces lo biológico emerge de lo social de manera descarnada, y remite a la complejidad de lo social. Voy a cerrar este apartado, desde el recuerdo como médico residente del Hospital de Niños Ricardo Gutiérrez de la ciudad de Buenos Aires en el año 1986, en la rotación por “Niño Sano”, donde los residentes de primer año atendían pacientes ambulatorios, y donde me correspondía, en tanto residente de tercer año, la coordinación de la tarea. Así llegó a la consulta una madre con su hijo de cuatro años, era una familia de bajos ingresos que vivía en el barrio de Palermo, en un departamento de un tercer piso al frente. En la consulta se detectó que las piernas del niño estaban arqueadas, y se consultó al servicio de nefrología que realizó un diagnóstico presuntivo de “raquitismo hipofosfatémico” (trastorno genético hereditario). Nos pareció tan extraño el diagnóstico que organizamos una visita a la casa de la familia para el día siguiente, a pesar de que la dirección del hospital no nos había autorizado la visita fuera del hospital. Al llegar, y sin entrar al edificio, cambiamos el diagnóstico presuntivo, por el de raquitismo por deficiencia de vitamina D, causado por la falta de exposición al sol, forma dominante e histórica de presentación del raquitismo. ¿Que habíamos descubierto?, que, si bien la familia vivía en Palermo, residía en un edificio “tomado”, en el que las ventanas estaban tapadas con chapas, dado que los balcones no tenían barandas, y por ende el sol no entraba al departamento. Paulo Freire señala que la cabeza piensa donde los pies pisan^76^.

## Territorios: potencia y complejidad

Los territorios negados en los procesos de vinculación entre Estados y profesionales, en los inicios de la modernidad^45^, deben ser reconocidos por las ciencias no como objetos de conocimientos, sino como espacios sociales con saberes propios, en diálogo y procesos de traducción con los conocimientos científicos^44^^,^^45^. Y en esas dinámicas, un concepto central a recuperar es el de potencia, formulado por Baruch Spinoza (1632-1677), quien no concibe la potencia como posibilidad, sino como una efectividad en acto, en la cual se persevera para afirmarse y desplegarse en la existencia, en la cual emerge el poder de afectar y de ser afectado^77^^,^^78^^,^^79^. Para Spinoza, la potencia no es un ideal de libre albedrío, sino un vivir según la razón, comprendiendo la necesidad^77^^,^^78^^,^^79^. 

Una pregunta que debemos hacernos es: ¿por qué pensamos en términos de poder y no en términos de potencia, cuando en los territorios encontramos sujetos y colectivos que se caracterizan por su potencia, y no por su poder? Ese sesgo, no es azaroso, y nos lleva a recuperar a Ludwig Wittgenstein (1889-1951) quien sostuvo que “los límites de mi lenguaje son los límites de mi mundo”, señalando que la comprensión, en tanto forma en la que experimentamos el mundo, está limitada por nuestro lenguaje. Y en la medida que el lenguaje se limita a lo instrumental, nos aleja de poder interpretar las multiplicidades y complejidades de lo territorial. De allí que el lenguaje técnico que estructura la razón instrumental no sea un buen camino para las transformaciones sociales en los territorios, o, mejor dicho, constituye la jaula de hierro que ya citamos. 

Boaventura de Sousa Santos postula que “el descredito de las soluciones no trae consigo el descredito de los problemas”. Para él, trabajar en los territorios requiere procesos de traducción, que deben basarse en el presupuesto de la imposibilidad de una teoría universal, por lo tanto, se debe crear inteligibilidad recíproca entre distintas experiencias, y entre los agentes y los colectivos en los territorios, en una deconstrucción que incluya tanto a los saberes como a las prácticas, para así identificar los residuos epistemológicos eurocéntricos que siguen vigentes, y reconstruir lo desplazado por el colonialismo y el neocolonialismo. Ese trabajo de deconstrucción no es solo técnico, sino también emocional, político e intelectual^44^.

## Del laberinto se sale por arriba

Las complejidades de los territorios exigen incorporar epistemologías concordantes con sus complejidades, que no pueden limitarse a lo instrumental^15^^,^^18^^,^^19^^,^^44^^,^^80^^,^^81^^,^^82^. De allí el título de este apartado que remite a la obra del poeta argentino Leopoldo Marechal (1900-1970).

Como hemos visto, los diseños industriales que siguieron las instituciones sociales simplificaron las estrategias para abordar las complejidades de los problemas en los territorios, reemplazando a los artesanos por obreros. Pero esto no consiguió camuflar el carácter ontológico de lo social que por esencia es relacional, y requiere de “artesanos lúdicos” y no de obreros. 

Trabajar en lo social es “hacer cosas con palabras”^83^^,^^84^^,^^85^, respetando los contextos, las culturas y los saberes de los territorios, de allí la imperiosa necesidad de incorporar marcos referenciales basados en lo interpretativo en reemplazo de lo explicativo^19^^,^^81^. Ese proceso implica, en los trabajadores, una fuerte tensión entre sus saberes y sus prácticas, lo cual debe ser pensado/discutido para trabajar la relación entre lo que se piensa, lo que se dice, y lo que se hace. La lógica fabril dominante en los campos sociales mecanizó el trabajo, las relaciones interpersonales y las dinámicas institucionales, perdiendo los colectivos la posibilidad de elucidación de su trabajo, en tanto “saber lo que se piensa y pensar lo que se hace”^86^.

El desafío que enfrentan tanto los directivos como los trabajadores y los territorios es el de cuidar la institucionalidad pública. Pero no basta con cuidarla, sino que hay que reinventarla, ya que las críticas sobre esa institucionalidad pública alcanzan al conjunto de los trabajadores, e incluso a los gremios que representan a esos trabajadores, y que reproducen las lógicas provenientes de la TGA^87^. Esto lleva a naturalizar la cosificación del otro (sean compañeros o usuarios), conformando institucionalidades basadas en el “sentido práctico”, en tanto un conocimiento sin conceptos^87^. 

Los cambios en los campos sociales exigen superar obstáculos epistemológicos^88^, que impiden entender y pensar esas instituciones y los territorios como complejos, inexactos, y humanos, y así construir una praxis que supere el “debe ser”, desde la concepción que “hacer es pensar”^23^. 

Se debe volver posible lo que se sostiene que es imposible. Y para ello debemos proponernos y construir que el trabajo en lo social no sea industrial, abandonando y superando la razón instrumental que anula los procesos de aprendizaje^2^^,^^89^^,^^90^, y a la vez habilitar lo lúdico como parte de las micropolíticas^80^^,^^83^^,^^91^^,^^92^, ya que el juego es el hilo conductor de la explicación ontológica^19^. Y en ese hacer jugando, debemos rescatar la pregunta que formuló León Rozitchner: ¿de qué manera volver a recuperar ese poder real que las instituciones al mismo tiempo que producen, expropian?^93^. 

Las nuevas institucionalidades sociales debieran caracterizarse por sus fuertes vínculos con los territorios; lo múltiple; la humanización de sus prácticas; la alta calidad de sus tareas; el predominio de lo relacional, lo artesanal y lo lúdico; ser bellas sin ser ostentosas; con equipos heterogéneos acordes a la complejidad y la impronta cultural y semiótica de los territorios. Nuevas institucionalidades en las que el aprender sea más importante que el enseñar; el cuidar se jerarquice sobre el atender; el territorio sobre la institución; la libertad sobre el encierro; lo relacional sobre lo racional; y la praxis sobre la teoría. Aspiramos a instituciones estructuradas sobre la base de las singularidades de lo territorial, y no de lo programático; donde el crecimiento institucional sea de tipo rizomático y no arbóreo^80^^,^^91^; donde lo humano se ubique sobre lo técnico, y lo social sobre lo artificial^2^. Teniendo en claro que el objetivo es el de estar juntos en los territorios, y no amontonados en grandes edificios^94^. Si bien todo lo anterior es fácil de escribir, pero difícil de hacer, y no podemos esperar resultados diferentes haciendo lo mismo. Del laberinto se sale por arriba.
